# TNFAIP8 regulates gastric cancer growth via mTOR‐Akt‐ULK1 pathway and autophagy signals

**DOI:** 10.1111/jcmm.16413

**Published:** 2021-03-08

**Authors:** Zheng Chen, Jianguo Zhang, Chenyang Dong, Dongsheng Li, Yuehan Yin, Wenhai Yu, Yuezhi Chen

**Affiliations:** ^1^ Tumor Research and Therapy Center Shandong Provincial Hospital Affiliated to Shandong First Medical University Jinan China; ^2^ Department of Gastrointestinal Surgery Liaocheng Dongchangfu People's Hospital Liaocheng China; ^3^ Department of Gastrointestinal Surgery Shandong Provincial Hospital Affiliated to Shandong First Medical University Jinan China

**Keywords:** autophagy, gastric cancer, mTOR‐Akt‐ULK1, TNFAIP8

## Abstract

In this study, the purpose of this study was to investigate the role of TNFAIP8 in gastric cancer (GC). The expression of TNFAIP8 was detected by RT‐PCR or western blot . TNFAIP8 was silenced or overexpressed in two cell lines. CCK‐8 assay, transwell assay and flow cytometry were used to analyse cell viability, cell invasion capability and apoptosis, respectively. Nude mice were inoculated with TNFAIP8 silencing or overexpressing cells to form transplanted tumours. HE staining and immunohistochemistry assay were performed to assess histopathological changes in tumours. We found that the mRNA and protein expression of TNFAIP8 were significantly up‐regulated in GC tumour tissues and cells compared with the normal counterparts. Overexpression of TNFAIP8 in GC cells increased cell viability, decreased apoptosis and promoted the cell migration ability. Meanwhile, increased expression of TNFAIP8 promoted autophagy, while inhibiting mTOR‐Akt‐ULK1 signal pathway. In conclusions, this study presents data that TNFAIP8 inhibits GC cells presumably by down‐regulating mTOR‐Akt‐ULK1 signal pathway and activating autophagy signal.

## 1 INTRODUCTION

Gastric cancer (GC) is one of the most common and fatal malignancies in the world. It is difficult to treat and easy to metastasize and spread. At the same time, poor prognosis is related to lymph node and peritoneal metastasis in GC, which is the main cause of GC recurrence.^1^ According to statistics, the number of new GC cases and cancer deaths in 2020 worldwide reached approximately 14.1 million and 8.2 million, respectively,^2^ while most of these cases occurred in developing countries. Like liver cancer and colorectal cancer in digestive system, GC posed a serious threat to human health. While the incidence of GC has decreased in the past few years, its mortality ranks second among all kinds of malignant tumours.^3^ Great progresses have been made in surgical resection and adjuvant therapy for GC, and early‐stage GC could be cured. However, most GC patients were diagnosed in the late stage, and originally effective treatment methods have become ineffective.^4^ Therefore, it is urgent to identify an effective tumour marker for early diagnosis and improvement of therapeutic effects.

As a new candidate oncoprotein, tumour necrosis factor‐α‐induced protein 8 (TNFAIP8) has gradually attracted the extensive attention.^5^ TNFAIP8, TNFAIP1 (TNF‐AINDUCEDPLOTEIN 8‐LIKE 1, TIPEL), TIPE2 and TIPE3F7L constitute a protein family.^6^ It has been shown that these family members with high homology differ in biological behaviours. Among them, TNFAIP 8 is the first member found in this family and was called GG2‐1, SCC‐S2 or MDC‐3.13.^7^ The TNFAIP 8 gene is located on chromosome 5q23.1 and expressed in most malignant foot tumour tissues. Studies on these tumours suggested that signal transduction pathway affects many processes such as cell apoptosis and therefore plays an important role in the formation and development of tumours.^8^ In recent years, multiple functions of TNFAIP 8 in tumour formation have been continuously identified. In these cases, TNFAIP 8 takes part in the process of apoptosis and autophagy in different types of cells, and overexpression of TNFAIP8 is frequently observed in malignant tumours.^9^ Moreover, TNFAIP8 overexpression is significantly correlated with excessive proliferation, reduced apoptosis, enhanced invasion and metastasis, as well as drug resistance. Previous studies have found that TNFAIP 8 expression could be detected in tumour tissues of various malignancies, such as breast cancer, colon cancer, ovarian epithelial cell cancer and prostate cancer.^10^, ^11^ However, the role of TNFAIP 8 in GC and the underlying mechanism remain to be further investigated.

## 2 MATERIALS AND METHODS

### 2.1 Reagents

The reagents used in the study were purchased as follows: RPMI‐1640 culture solution from Gibco Company of the United States; foetal bovine serum and double antibody from Thermophilic Technology Company; CCK‐8 kit from Japan Tongren Chemical Company; Transwell chamber and artificial basement membrane from BD company in USA; the flow apoptosis detection kit from Nanjing Keygen Biology Co., Ltd; antibodies against LC‐3I/LC‐3II (#12741, 1:1000), P62 (#23214, 1:1000), p‐Akt (#4060, Ser 473, 1:1000), Akt (#4685, 1:1000), p‐mTOR (#5536, Ser2448,1:1000), p‐ULK1 (#14202, Ser757,1:1000), ULK1 (#6439, 1:1000), and GAPDH (#5147, 1:1000) from Cell Signaling Technology; antibodies against TNFAIP8 (ab251212, 1:1000), Bad (ab32445, 1:1000), Bax (ab32503, 1:1000) and Bcl‐2 (ab182858, 1:1000) from Abcam. 3‐Methyladenine (3‐MA, #M9281) from Sigma; and 3‐benzyl‐5‐((2‐nitrophenoxy) methyl)‐dihydrofuran‐2 (3‐BDO, #R193885) from Aladdin.

### 2.2 Collection of gastric cancer tissues and normal tissues

A total of 87 pairs of GC tissues and corresponding adjacent non‐tumorous tissues were obtained from Shandong Provincial Hospital Affiliated to Shandong First Medical University between 2013 and 2018. No local or systemic treatment was conducted in these patients before the operation. This study was approved by the Research Ethics Committee of Shandong Provincial Hospital Affiliated to Shandong First Medical University. Informed consents were obtained from all patients.

### 2.3 Animals

A total of 24 male BALB/C (nu/nu) nude mice were purchased from Shanghai Sciple‐Bikai Experimental Animal Co., Ltd. (the quality certificate number of SCXK (Hu) 2019‐0016). The mice were aged 4 weeks, weighted 15 g and raised under SPF conditions. All operations were carried out in accordance with the animal ethics regulations of Shandong Provincial Hospital Affiliated to Shandong First Medical University.

### 2.4 Cell culture

Cell lines GES‐1, SGC‐7901, NCI‐N87, MKN‐28 and MGC‐803 were purchased from ATCC (USA). The cells were maintained in RPMI‐1640 culture solution containing 10% foetal bovine serum and cultured in 5% CO_2_ incubator at 37℃. Cells were digested and sub‐cultured with 0.05% pancreatin after reaching 70%‐80% confluence at the adherent growth.

### 2.5 RT‐qPCR assay

Cell lines GES‐1, SGC‐7901, NCI‐N87, MKN‐28 and MGC‐803 were cultured and collected for detection of TNFAIP8 expression. Total RNA was extracted from the cultured cells using Trizol reagent and then reversely transcribed into cDNA using cDNA reverse transcription kits. RT‐qPCR reaction was carried out on StepOnePlus real‐time fluorescence quantitative PCR system by the two‐step method. The reaction conditions were as follows: pre‐denaturation at 95°C for 1 minutes, followed by 40 cycles of denaturation at 95°C for 5 s, annealing and extension at 60°C for 30 S.

### 2.6 Cell viability

GC cells with knockdown or overexpression of TNFAIP8 were inoculated into 96‐well plates at a density of 5000 cells per well. The cells were cultured respectively for 24, 48 and 72 hours, and then 10 µL of cell counting kit‐8 (CCK‐8) solution (5 mg/m) was added to each well for 4 hours of incubation. Thereafter, 150 μl of DMSO solution was added to each well, and then mixed on a shaker at a low speed for 10 minutes. Finally, the absorbance was detected at 490 nm on a microplate reader.

### 2.7 Establishment of human GC mouse model

While nude mice were adaptively fed for 1 week, human GC MKN‐28 cells were infected with TNFAIP8‐shRNA lentiviruses (sh‐TNFAIP8) or lentiviruses overexpressing TNFAIP8 (Lv‐TNFAIP8). After infection, cells were cultured in logarithmic growth phase, collected and centrifuged at 800 rpm for 4 minutes. Following removal of the supernatant, a single‐cell suspension at a concentration of 2 × 10^7^ cells/ml was prepared. Thereafter, 0.2 ml of the cell suspension was inoculated into each nude mouse under the armpit in a sterile environment, and the inoculated mice were then put back into the cage for feeding and observation. Total tumour volume was measured every four days. All animals were sacrificed 28 days after inoculation, and tumour masses were weighed and used for subsequent molecular analyses.

### 2.8 Cell transfection

Cells were seeded into 6‐well plates 1 day before transfection. Transfection was performed on adherent cells with a fusion rate of 40% to 60%. The original medium was removed during transfection, and the cells were washed twice with serum‐free RPMI1640 medium. 4 μL of lipofectamine 2000 liposomes was added to 500 μL of serum‐free RPMI1640 culture solution, while 10 μL of TNFAIP8 mimics storage solution was added to another 500 μL of serum‐free RPMI1640 medium. After 5 minutes of standing at room temperature, the above two solutions were mixed and allowed to stand at room temperature for 20 minutes. The mixed solution was then added to the cells, and the final concentration of mimics was 80 nmol/L. After 6 hours of incubation, the DMEM medium containing 10% foetal bovine serum was replaced, and the culture was continued for subsequent experiments.

### 2.9 Transwell assays

The small chamber for Transwell assay was put into a culture plate. 300 μl of pre‐heated serum‐free culture medium was added into the upper chamber. After 15‐30 minutes of standing at room temperature for rehydration of the matrix glue, the remaining culture solution in the upper chamber was sucked off, and a cell suspension of 5 × 10^5^ cells /ml for each group was prepared using a serum‐free medium with bovine serum albumin (BSA). While 100‐200 μL of cell suspension was added into Transwell upper chamber, 500 μl of culture medium containing 20% FBS or chemokines was applied to the lower chamber. Thereafter, cells at the bottom of the lower chamber were stained with 0. 5% crystal violet, while those at the inner side of the upper chamber were removed with cotton swabs. Finally, cell counting and morphological analysis were performed under a light microscope.

### 2.10 Flow cytometry

Cells stably transfected with TNFAIP8, cells stably transfected with an empty vector and non‐transfected cells were inoculated respectively on a 96‐well plate at a density of 2 × 10^4^ cells/well. Then, the cells were collected and analysed using an apoptosis detection kit according to the manufacturer’s instructions. And the cells were detected by flow cytometer immediately after the colour was dyed. 10 μM 3‐MA (PI3K inhibitor) dissolved in DMSO and 10 μM 3‐BDO (MTOR kinase activator) dissolved in DMSO were used in the experiments. Annexin V staining positive cells were early apoptotic cells and PI staining positive cells were necrotic cells, and annexin V and PI staining were double positive. The cells were late apoptotic cells, and annexin V and PI staining were double negative.

### 2.11 Ki‐67 staining

Tumour tissue specimens were fixed with 4% formaldehyde and embedded in paraffin. All tissues were cut into 4 μm‐thick sections using a German thermoscience cryostat, and the sections were then incubated with primary Ki‐67 antibody. Thereafter, the sections were washed with PBS and subjected to an incubation with secondary antibody according to the manufacturer's instructions.

### 2.12 Western blotting

The samples were minced, homogenized in ice‐cold RIPA buffer containing 2 mM PMSF, and then centrifugated at 12 000 g for 15 minutes at 4°C. The total protein concentration was measured by using a BCA assay kit. The proteins were subjected to SDS‐PAGE electrophoresis and transferred to PVDF membranes. After being blocked in 5% skim dried milk for 2 hours, membranes were incubated with primary antibodies at 4 °C overnight, followed by an incubation with HRP‐conjugated secondary antibody for 2 hours. Protein bands were visualized by using an enhanced chemiluminescence (ECL) advanced kit and a gel imaging system (Tanon Science & Technology Co., Ltd., China).

### 2.13 Statistical analysis

All data were presented as mean ± SD. The statistical significance of differences between the means of each group was analysed by one‐way ANOVA followed by Tukey multiple comparison tests. Student’s t test was used to make a comparison between two groups. A p value less than 0.05 was considered statistically significant.

## 3 RESULTS

### 3.1 TNFAIP8 expression in gastric mucosal cells and GC cells

**FIGURE 1:**
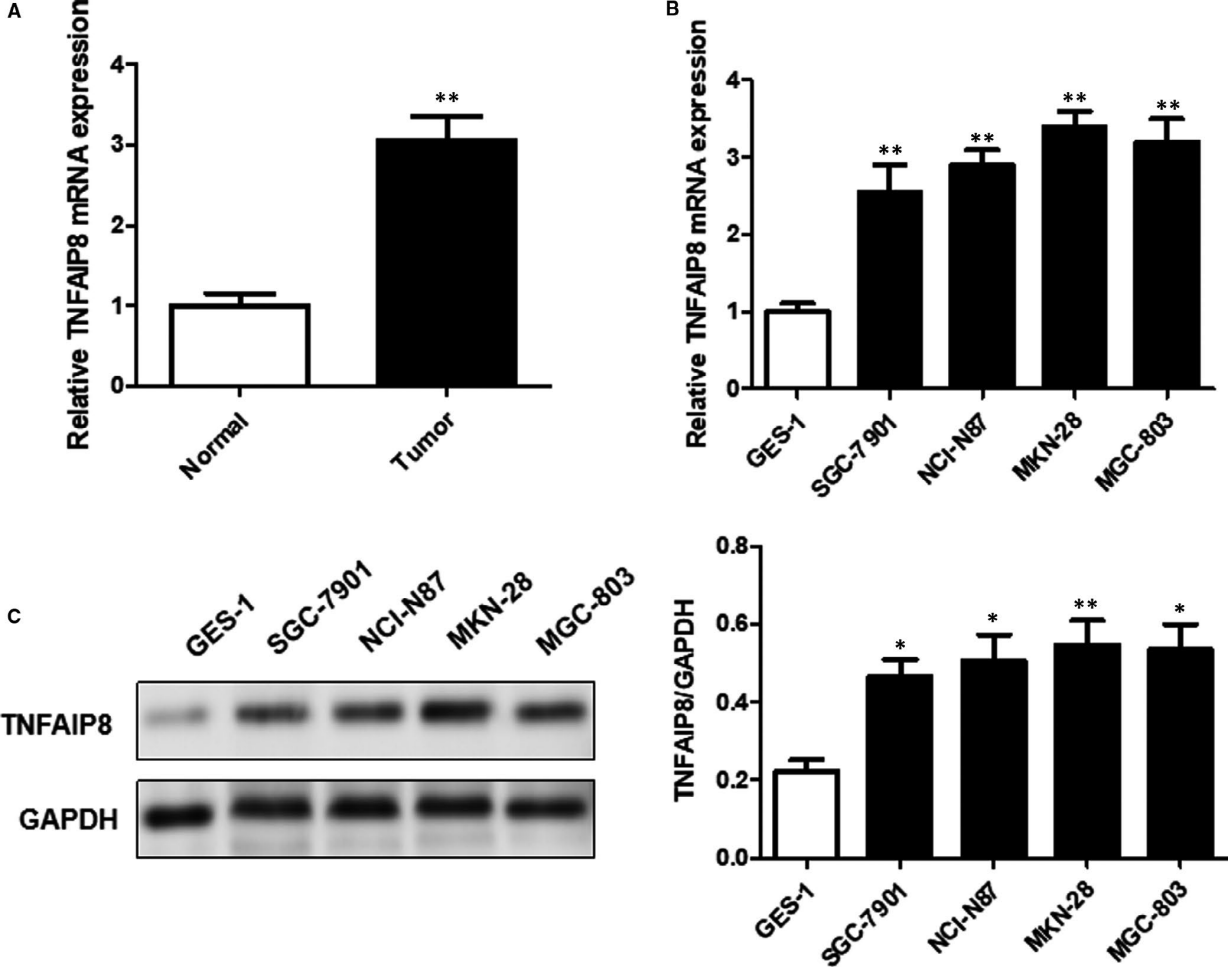
Figure 1 TNFAIP8 expression was up‐regulated in GC tissues and cells. (A) Relative mRNA expression of TNFAIP8 expression in GC tissues and matched normal tissues were determined using RT‐PCR. (B) TNFAIP8 mRNA (B) and protein (C) expression levels in GC cells and human gastric mucosa epithelial cell line were also determined using RT‐PCR or western blot. Data are expressed as the mean ± SD. Compared with normal or GES‐1: *p < 0.05 and **p < 0.01

As depicted in Figure 1A, GC tissues displayed a significantly higher level of TNFAIP8 mRNA expression than the normal counterparts. Likewise, a marked increase in the expression of TNFAIP8 at both mRNA and protein levels was detected in GC cell lines SGC‐7901, NCI‐N87, MKN‐28 and MGC‐803 compared with the normal gastric mucosa cell line GES‐1 (Figure 1B and 1C).

### 3.2 Overexpression of TNFAIP8 in GC cells increased cell viability, reduced apoptosis and enhanced the cell migration ability

**FIGURE 2:**
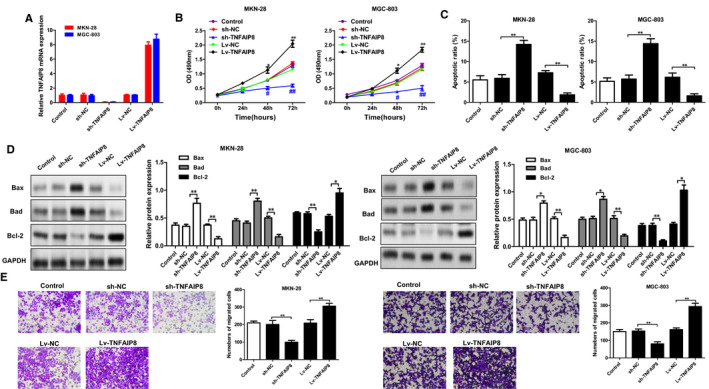
Overexpression of TNFAIP8 increased cell viability, reduced apoptosis and enhanced the cell migration ability. The mRNA of TNFAIP8 was determined in GC cells after si‐XIST or Lv‐TNFAIP8. (B) Cell viability was measured using the CCK‐8 assay. (C) Flow cytometry was used to detect cell apoptosis. (D) The apoptosis related protein was determined by western blot. (E) Transwell assay was used to detect the cell migration ability. Data are expressed as the mean ± SD. Compared with the control: *p < 0.05 and **p < 0.01

As shown in the PCR assay (Figure 2A), knockdown and overexpression of TNFAIP8 gene were successfully performed in MKN‐28 and MGC‐803 cells. Notably, TNFAIP8 overexpression obviously increased cell viability and reduced apoptotic rate as well as the expression levels of Bax and Bad, while enhancing the cell migration ability and Bcl‐2 expression. Conversely, knockdown of TNFAIP8 gene remarkably decreased cell viability and increased the apoptotic rate as well as the expression levels of Bax and Bad, while reducing the cell migration ability and Bcl‐2 expression (Figures 2B‐E).

### 3.3 TNFAIP8 overexpression increased tumour volume and weight in mice with transplanted tumour

**FIGURE 3:**
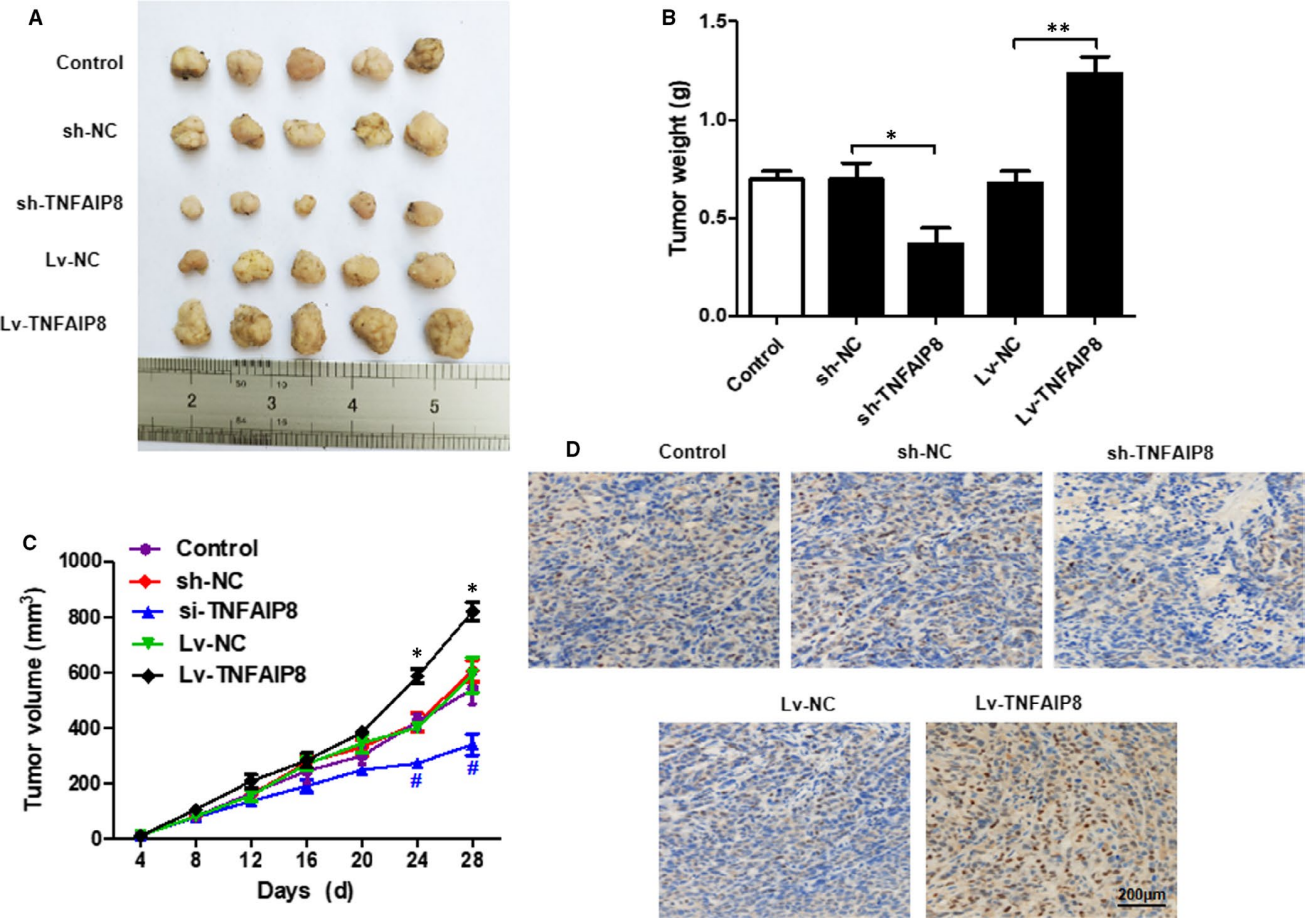
Overexpression of TNFAIP8 increased tumour volume and weight in mice with transplanted tumour.(A, B) Mice were killed and tumor weight was examined in each group. (C) Tumor volume was calculated every 4 days. (D) The expressions of Ki67 were detected by immunohistochemistry. Data are expressed as the mean ± SD. Compared with LV‐NC or Sh‐NC: *p < 0.05 and **p < 0.01

As depicted in Figures 3A‐C, significantly increased tumour volume and weight were observed in mice with transplanted tumour overexpressing TNFAIP8 gene compared with the control mice, while silencing TNFAIP8 gene led to a marked reduction in tumour volume and weight of mice with transplanted tumour. In the meantime, Ki67 immunohistochemical analysis showed that the number of positive cells with overexpressed TNFAIP8 gene was significantly increased, whereas the number of positive cells with silenced TNFAIP8 gene was significantly reduced (Figure 3D).

### 3.4 TNFAIP8 overexpression promoted autophagy and inhibited mTOR‐Akt‐ULK1 signalling pathway

**FIGURE 4:**
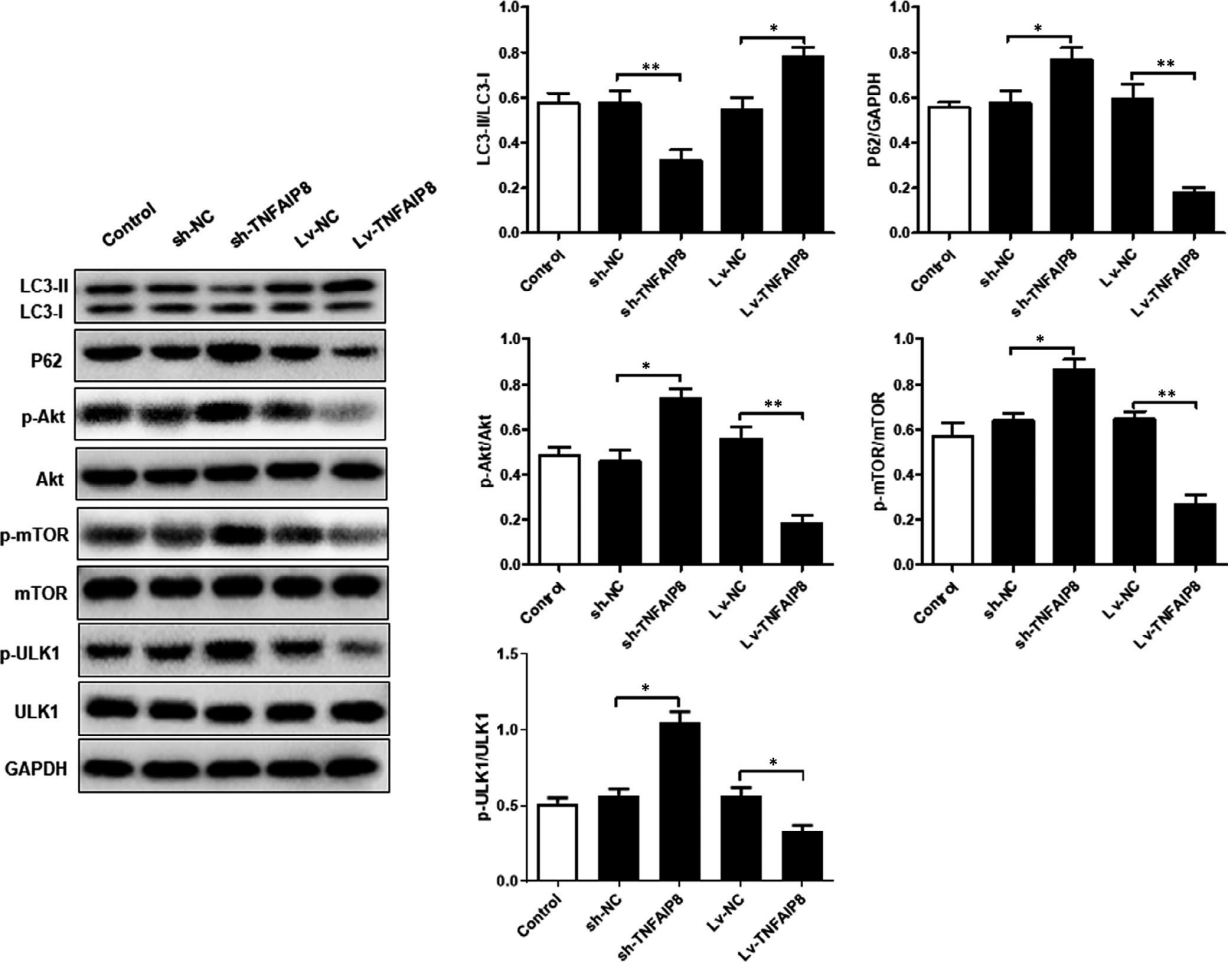
Overexpression of TNFAIP8 promoted autophagy and inhibited mTOR‐Akt‐ULK1 signalling pathway. Data are expressed as the mean ± SD (n = 5). Compared with LV‐NC or Sh‐NC: *P < 0.05 and **P < 0.01

As shown in Figure 4, TNFAIP8 overexpression significantly elevated the ratio of LC3I/LC‐3II and P‐ULK1 levels, while decreasing the expression levels of P62, P‐PI3K and P‐mTOR. By contrast, knockdown of TNFAIP8 gene led to a significant decrease in the ratio of LC3I/LC‐3II and P‐ULK1 levels as well as an elevation in the levels of P62, P‐PI3K and P‐mTOR.

### 3.5 TNFAIP8 regulates autophagy‐related signals

**FIGURE 5:**
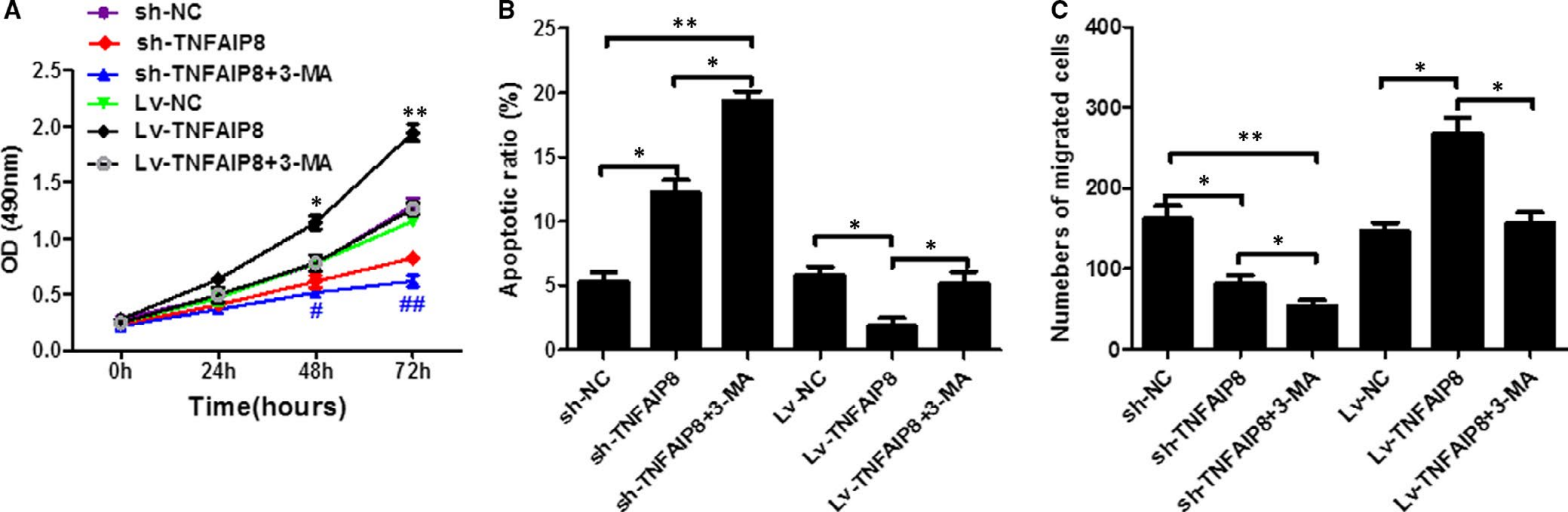
TNFAIP8 regulates cell apoptosis and migration ability. (A) The Cell viability was measured using the CCK‐8 assay. (B) Flow cytometry was used to detect cell apoptosis. (C)Transwell assay was used to detect the cell migration ability. Data are expressed as the mean ± SD. Compared with LV‐NC or Sh‐NC: *p < 0.05 and **p < 0.01

**FIGURE 6:**
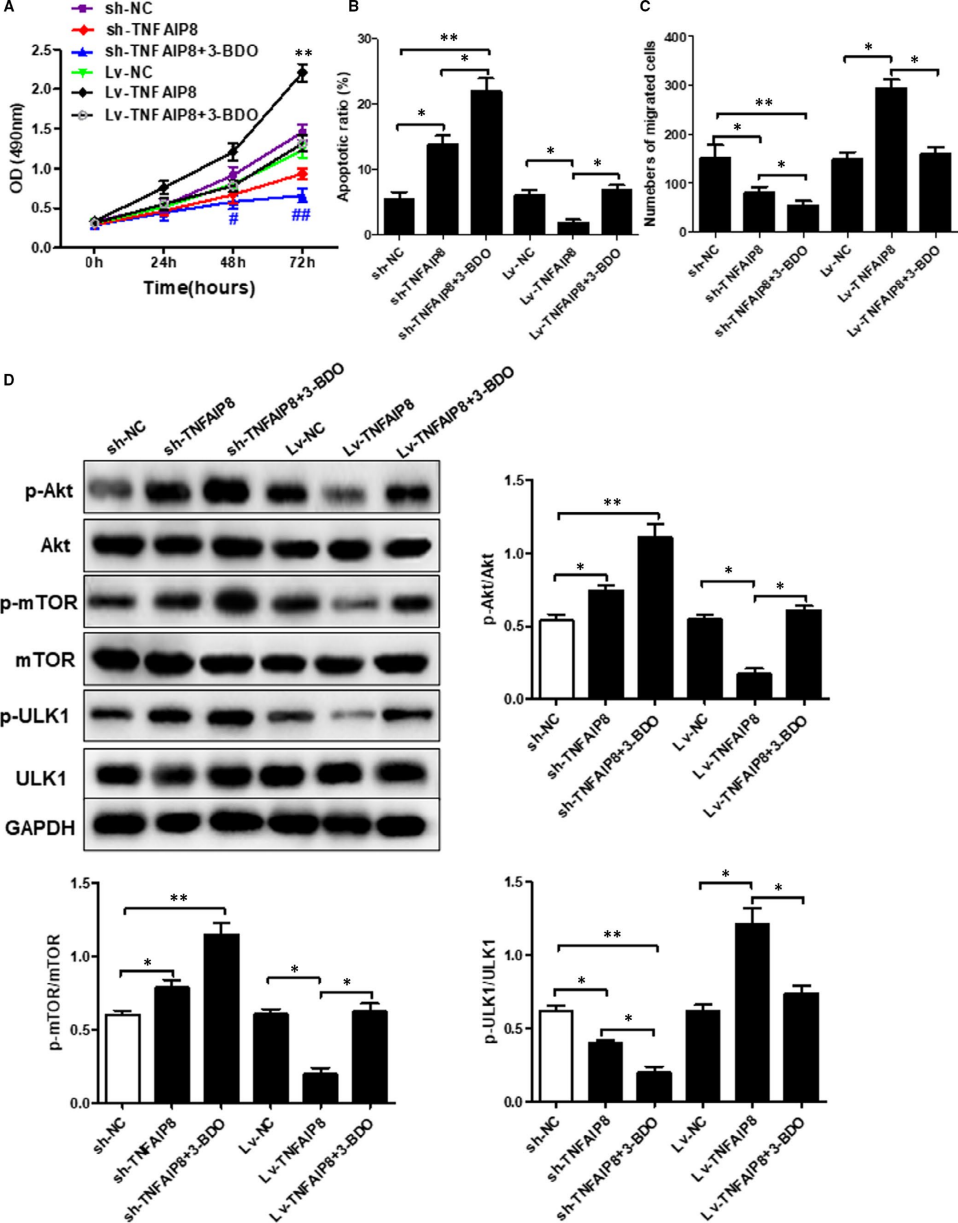
The role of TNFAIP8 in regulation of autophagy signals. (A) The Cell viability was measured using the CCK‐8 assay. (B) Flow cytometry was used to detect cell apoptosis. (C)Transwell assay was used to detect the cell migration ability. (D) The protein expression was detected by western blot. Data are expressed as the mean ± SD . Compared with LV‐NC or Sh‐NC: *p < 0.05 and **p < 0.01

As shown in Figure 5, overexpression of TNFAIP8 significantly increased cell viability, decreased apoptotic rate and enhanced the cell migration ability. Strikingly, the above TNFAIP8 overexpression‐caused effects were remarkably reversed by the autophagy inhibitor or autophagy activator. Moreover, the autophagy activator reversed the altered expression of P‐P3K, P‐mTOR and p‐ULK1 caused by TNFAIP8 overexpression (Figure 6).

## 4 DISCUSSION

As a common malignancy of the digestive system, GC poses a serious threat to human health like liver cancer and colorectal cancer.^12^ In China, the current incidence and mortality rate of GC are 29.9 per 100 000 (male 41.3/100 000 vs. female 18.5/100 000) and 22.3 per 100 000 (male 30.1/100 000 vs female 14.6/100 000), respectively.^9^ The current treatment method for GC is comprehensive surgery (including adjuvant chemotherapy, molecular report drug treatment).^13^ To date, great progresses have been made in surgical resection and adjuvant chemotherapy for GC, while early‐stage GC can be cured.^9^ However, most of GC patients are diagnosed in an incurable stage. In these cases, originally effective treatments have become ineffective, leading to a poor overall prognosis. It has been suggested that detection of EGF, cycinE, p27, CD44v6, MMP‐1, TIMP‐1, HER‐2, HER‐3 and VEGF may have a significance for the individualized treatment of GC patients.^9^ So far, while the pathogenesis of gastric cancer has yet to be fully elucidated, there are no effective indicators for early diagnosis, early treatment, improved prognosis and prolonged survival of patients in GC. Therefore, it is urgent to identify an effective tumour marker for early diagnosis and to improve the treatment effect of GC.

As a new candidate oncoprotein, tumour necrosis factor‐α‐induced protein 8 (TNFAIP8) has recently attracted widespread attention.^14^ It has been shown that TNFAIP8 plays important roles in the formation and development of tumours, while being involved in the regulation of cell proliferation, tumour invasion, migration, death and drug resistance in different tumour types. Xing et al. investigated the expression of TNFAIP8 in non‐small cell lung cancer tissues and adjacent normal lung tissues.^15^ Miao et al. analysed the expression pattern of TNFAIP8 in 92 colon cancer tissues by immunohistochemistry and found that TNFAIP8 was overexpressed in 48.9% of the patients (45/92). To date, the expression of TNFAIP8 in GC and its role in regulating the growth, invasion and migration of GC cells remain to be determined. In the present study, PCR assay revealed that TNFAIP8 expression was significantly increased in GC tissues compared with the corresponding normal tissues. In addition, we observed that the four GC cell lines displayed a significantly higher level of TNFAIP8 expression than normal gastric mucosa‐derived cell line. These results indicate that TNFAIP8 is involved in GC pathogenesis.

At present, there are few studies on the role of TNFAIP8 in GC, and it is hard to determine whether down‐regulation of TNFAIP8 will affect the biological characteristics of GC cells. In the first experiment, we have shown that while TNFAIP8 was expressed in both GC tissues and cells, GC tissues and cells had a higher level of TNFAIP8expression than the corresponding paracancerous tissues and normal gastric mucosal cells, respectively. All these findings suggest that TNFAIP8 may be closely related to the occurrence and development of GC.

Autophagy is a type of apoptotic pathway‐independent cell death, which has become a research hotspot. While autophagy can play an anti‐tumour and anti‐ageing effect, it provides cells with nutrients to help them survive the harsh environment and has promoting effects on the development of tumours.^16^ Thus, autophagy is a ‘double‐edged sword’ in the tumorigenesis and tumour development. Hypoxia, ischaemia and radiation therapy can promote autophagy in tumour cells, tumour tissue lacks blood supply, and metastasis is prone to place tumour cells in a metabolic stress state.^17^ In this study, we found that overexpression of TNFAIP8 significantly increased cell viability, decreased apoptotic rate and enhanced the cell migration ability, while these alterations caused by TNFAIP8 overexpression were significantly reversed by autophagy inhibitor and activator. The above observation suggests that TNFAIP8 may exert vital effects on tumour cells by activating autophagy signals and regulate the pathophysiology of GC.

Akt is a key downstream molecule of PI3K and binds to PI3K via its N‐terminal PH domain. While Akt can regulate the proliferation and survival of many types of cells,^18^ it acts as a key regulator of multiple cellular processes, such as cell proliferation, differentiation and survival. mTOR is a large protein with a C‐terminal serine/threonine PI3K‐related kinase domain and acts as a main substrate of ATK. As an important cell signal transduction pathway, mTOR signalling is involved in physiological activities including cell growth, survival and autophagy. Studies have shown that abnormal mTOR signalling exerts profound effects on cell homeostasis and may even lead to the development of pathological conditions, such as GC.^19^ Tian et al conducted immunohistochemistry to detect the expression of AKT in 128 pairs of GC tumour tissues and adjacent non‐tumour tissues, and found that the tumour tissues displayed a significantly higher level of AKT expression than adjacent non‐tumour tissues, and the high expression of AKT in GC was related to the T stage.^20^ It was reported that the expression of phosphorylated AKT (phospho‐AKT, p‐AKT) in tumour tissues was detected in 119 out of 231 patients with gastric cancer (53%). Statistical analysis showed that p‐AKT expression was associated with poor prognosis of the patients. Li et al. analysed the expression of mTOR in 33 GC patients and 30 healthy controls by immunohistochemistry and found that mTOR was expressed in 51.5% of the patients, while there was almost no mTOR expression in the controls. The above observations suggest that mTOR activation may occur during the occurrence and development of GC.^21^ In addition, the above study found that mTOR expression was associated with late tumour stage, poor differentiation and lymph node metastasis. ULK1 (Unc‐51 Like Autophagy Activating Kinase 1) complex initiates the formation of autosome and could be regulated by mTORC1 and MAPK‐related kinases. mTORC1 integrated growth factors, regulated oxygen content, amino acids and energy, and promoted the synthesis of proteins related to cell growth and metabolism.^22^ Upon activation, mTORC1 reduced the activity of ULK1 kinase by phosphorylating ULK1 and ATG13. In the present study, we investigated the relationship between the TNFAIP8 and the mTOR‐Akt‐ULK1signalling pathway as well as its role in gastric cancer. Our results showed that overexpressed TNFAIP8 significantly reduced the levels p‐Akt and p‐mTOR, while increasing p‐ULK1 level. Conversely, knockdown of TNFAIP8 markedly up‐regulated p‐Akt and p‐mTOR, while significantly down‐regulating p‐ULK1. Notably, the above alterations were significantly reversed by autophagy activators. Collectively, these results indicated that TNFAIP8 inhibits the mTOR‐Akt‐ULK1 and is involved in physiological and pathological processes of GC.

## 5 CONCLUSION

In summary, this study found that TNFAIP8 was highly expressed in GC tissues, while significantly inhibiting the mTOR‐Akt‐ULK1 signalling pathway and activating autophagy signal. These findings may contribute to a better understanding of GC pathogenesis and potentially provide a new therapeutic target for GC.

## CONFLICTS OF INTEREST

There are no conflicts of interest to declare.

## AUTHORS’ CONTRIBUTIONS

ZC, JGZ, CYD and YZC: performing the experiments, data analysis and writing the paper. DSL, YHY and WHY: designing the present study and providing experimental materials. All authors read and approved the final manuscript.

## ETHICAL APPROVAL

All the experimental procedures were approved and conducted in accordance with the Institutional Animal Care and Use Committee of Shandong Provincial Hospital Affiliated to Shandong First Medical University.

## Data Availability

The data used to support the findings of this study are included in the article.
